# Modern Sociology

**Published:** 1904-01-23

**Authors:** 


### MODERN SOCIOLOGY.

### IS OUR CIVILISATION A FAILURE?

"Is our civilisation a failure, and is the Caucasian played out?" asked "Truthful James" more years ago than many among us care to count. Without venturing on an answer to the former question, one can hardly evade a reply of some kind to the latter, when one reads the statistics collected by the Registrar-General. All over Europe, as it seems, the birth-rate is decreasing, sometimes along with a decreasing marriage-rate, sometimes in face of it, but everywhere decreasing. The progress in public health is satisfactorily shown throughout our continent by a diminishing death-rate, but unless births maintain their old figures while deaths diminish, the nation with which they have to do is not increasing in vitality. Nay, as some of the lives preserved are those of the comparatively sickly who would have died under an earlier and harder régime, a decreasing death-rate, when coupled with a decreasing birth-rate, serves but to show a diminution of the vitality of the race.

The figures available for the different countries of Europe begin at different periods, but for the majority they are available from the year 1872, the year which may be remembered in Britain as that which witnessed the passing of the Elementary Education Act. Earlier statistics are available for our own country, but the annals of 30 years are enough to show the trend of public sentiment. If one could deduce the birth-rate from the marriage-rate, it would be easy to lay the fault of the paucity of both upon national prosperity, but they do not increase and diminish *pari passu*. Thus in the United Kingdom we had, in 1872, a marriage-rate of 15·9 per thousand coincident with the birth-rate of 34·3, which rather increased during the two succeeding years. The birth-rate never fell below 34 per thousand until 1879, although the marriage-rate declined. In that year the marriage-rate was 13 3 per thousand, while the birth-rate was 33·3. In 1901, the last year for which statistics are available, the marriage-rate was 15·1, and the birth-rate only 28 per thousand, a proportion which had varied but little since 1896, and that only in the direction of an increased marriage and a decreased birth-rate. France has long been the "awful example" of statisticians in this matter, and not without reason, though the troubles through which France passed during 1870 and 1871 may reasonably explain a low marriage-rate without falling back for excuse on the old French proverb, "Il faut faire le pot avant de faire l'enfant." Still, the marriage-rate in 1872, while the ravages of the Franco-German war were still perceptible, was 19 5 per thousand, and the birth-rate 26·7, while in 1901, after nearly thirty years of peace and increasing prosperity, the marriage-rate was 15·6 per thousand, and the birth-rate 22. It is, however, matter for congratulation that both show an increase since 1898.

But what of Germany, the protagonist of France in 1870? In 1872 its marriage-rate stood at 21·6 per thousand, and its birth-rate at 39·5. It says something for the virility of the German nation that in the succeeding years up to 1877, although the marriage-rate fell the birth-rate increased to more than 40 per thousand, but since then the latter has shown a steady decrease. In 1901 the marriage-rate stood at 16·5 per thousand, and the birth-rate at 35·7. This is very much better than Britain's record, but it shows in a smaller degree the same tendency to degeneracy.

Belgium has much the same tale to tell as France. In 1872 the marriage-rate stood at 15·5 per thousand and the birth-rate at 32·3, proportions which had been exceeded only by a decimal point since 1853. But in 1901 the marriage-rate had risen to 16·6, while the birth-rate had fallen to 29·4. The Netherlands make rather a better show. There the marriage-rate in 1872 was 16·5 and the birth-rate 36 per thousand, while in 1901 the marriage-rate was 15·4 and the birth-rate 32 3, the former, however, showing an increase in the figures every year since 1878, while the latter declined from 33 2 in 1889, and since then has never been more than a fraction over 32, and sometimes even less. In the other northern kingdoms the same conditions, though less marked, still exist, with the exception of Norway, where in 1872 the marriage-rate stood at 14 per thousand and the birth-rate at 29·7, while in 1901, with a slightly lower proportion of marriages—13·4 per thousand—the birth-rate stands at 29·8. But it is doubtful if, in spite of its popularity with tourists, Norway as a whole is as prosperous as Sweden, where manufactures are commoner, or Denmark, whose agricultural position has improved so greatly within recent years. And in Sweden a marriage-rate of 13·9 in 1872 has given place in 1901 to one of 12·1, and a birth-rate of 30 per thousand to one of 26 8; while in Denmark a marriage-rate of 15 and a birth-rate of 30·3 has fallen within the 30 years to a marriage-rate of 14·4 and a birth-rate of 29·9. The declension here is not so marked, but is noteworthy because it has accompanied—as Danes themselves will tell us—an increase in general wealth.

In the Romance nations the same phenomenon is to be seen. In Italy a marriage-rate of 15 and a birth-rate of 37·9 per thousand has given place to a marriage-rate of 14 5 and a birth-rate of 32·6. For Spain there seem to be no statistics available between 1870 and 1878, but if we take 1870 as representing the earlier period, we find that in that year the marriage-rate was 12·4 per thousand and the birth-rate 35 5, while in 1901 the marriage-rate had risen to 16 9 while the birth-rate had fallen to 34·7. From the point of view of more northerly nations these countries are still reasonably prolific, but each shows the same phenomenon of a decreasing birth-rate, even when the proportion of marriages is increasing. Yet it can hardly be said that the economic condition of either of these countries has been declining during the time, although economists are always inclined to associate the variations of the marriage-rate with corresponding variations in the wealth of the people. The obvious explanation—though it does not fit all the cases—is the comparative postponement of marriage to a maturer age than was common formerly. But beyond that lies the question—Why this postponement? One reason undoubtedly is the development of industrialism in those fields where women's labour is preferred—for cheapness or any other reason—to that of men. Marriage is no longer women's one profession, even though it may be her ultimate destiny, and very rarely is she willing to accept it until she has tasted the bitter as well as the sweets of independence. Moreover, in every class, from the highest to all but the lowest—where alone the marriage of boys and girls is common—the standard of living has risen, often out of all proportion to the opportunities for satisfying its demands. This is not a thing wholly to be deplored. It is by trying to meet the demands of men that civilisation moves on its way. But there are certain facts that civilisation does not touch—and the laws of reproduction are among them. And while Europeans are talking of the "Yellow Peril" and Americans are watching anxiously the increase among the coloured inhabitants, with which the white population fails to keep pace, it may be worth while to remember Napoleon's cynical saying that Providence is always on the side of the big battalions—a saying which remains true even when the battalions are infantile.

